# Time trend of respiratory viruses before and during the COVID‐19 pandemic in severe acute respiratory virus infection in the Sultanate of Oman between 2017 and 2022

**DOI:** 10.1111/irv.13233

**Published:** 2023-12-13

**Authors:** Hanan Al Kindi, Luke William Meredith, Amina Al‐Jardani, Fathima Sajina, Intisar Al Shukri, Rehan al Haj, Fatma Alyaquobi, Adil Al Wahaibi, Amal Al Maani

**Affiliations:** ^1^ Central Public Health Laboratories, Ministry of Health Directorate General for Disease Surveillance and Control Muscat Oman; ^2^ Department of Health Emergencies World Health Organization Regional Office for the Eastern Mediterranean, Infectious Hazard Management Cairo Egypt; ^3^ Department of Control, Ministry of Health Directorate General for Disease Surveillance and Control Muscat Oman; ^4^ Department of Surveillance, Ministry of Health Directorate General for Disease Surveillance and Control Muscat Oman; ^5^ Ministry of Health Directorate General for Disease Surveillance and Control Muscat Oman

**Keywords:** COVID‐19, laboratory, respiratory, SARI/ILI, surveillance

## Abstract

**Introduction:**

Severe acute respiratory illness (SARI) is a potentially lethal condition, necessitating thorough medical care. COVID‐19 underscored the SARI threat, but other high‐risk pathogens require monitoring alongside SARS‐CoV‐2. Oman instituted a comprehensive testing system to gauge the prevalence of these pathogens between 2017 and 2021, aiding resource allocation and public health responses to potential respiratory pathogen outbreaks.

**Methods:**

Samples from SARI cases admitted to ICU were tested for pathogens using the Fast‐Track Diagnostic (FTD) molecular assay, a respiratory virus panel (RVP) that tests for 21 pathogens, including 20 viruses, by qPCR.

**Results:**

Between 2017 and 2022, ~30 000 samples were analysed using the RVP panel. Among SARI patients, 8%–42% tested positive for respiratory pathogens, with 4% showing multiple infectious agents, especially in children under 10. A drop in positivity during 2020–2021 can be attributed to SARS‐CoV‐2 control measures, followed by a rebound in infections in early 2022.

**Discussion:**

The COVID‐19 pandemic heightened awareness of respiratory pathogens' spread without adequate control measures. Influenza A/B, human rhinoviruses and respiratory syncytial virus constituted over 50% of severe acute respiratory illness cases in Oman over the past 5 years. During the pandemic, the incidence of these infections significantly declined, demonstrating the efficacy of COVID‐19 prevention measures in reducing spread of other pathogens.

## 1 INTRODUCTION

Since late 2019, the COVID‐19 pandemic has been circulating the globe, demonstrating the risk of respiratory pathogens to the international population. Over 750 million cases have been recorded since the pandemic was declared in January 2020, resulting in over 6.5 million deaths,^1^ making it one of the worst recorded pandemics in history. Respiratory viruses present a continual and ongoing threat, due to the ease of transmission through either droplet‐borne or airborne absorption into the respiratory tract, and can result in severe diseases of both the upper and lower respiratory tract.^2^ The treatment options are often limited, with therapeutics for viruses other than influenza being limited, meaning symptomatic treatment is often the only option, including providing oxygen, through respirators in extreme cases, until the patient recovers.^3^

Prior to the COVID‐19 pandemic, there was a general awareness of influenza as a respiratory pathogen, with programmes implemented to monitor circulating strains and variants on an annual basis. The Global Influenza Surveillance and Response System (GISRS) and National Influenza Centres work together to provide ongoing monitoring of influenza.^4^ This network also monitors other common respiratory pathogens, such as respiratory syncytial virus (RSV), a pathogen that mostly affects children, immunocompromised or elderly patients, but can have severe pathology resulting in death in up to 3% of cases,^5^ particularly when the patient is immunocompromised, young or elderly, or has an underlying condition. Rhinoviruses, which are the most common causative agent of the common cold,^6^ particularly in children, have extreme social and economic impacts on countries resulting in children and adults being hospitalised or being off work for long periods of time. Other pathogens such as parainfluenza^7^ and *Mycoplasma pneumoniae* ^8^are common nosocomial infections in children and immunocompromised or elderly patients, while there are a number of coronaviruses^9^ also associated with disease that have severe impacts on patient health and well‐being.

The Sultanate of Oman has long recognised the threat that these pathogens present and has a robust sentinel surveillance programme in place for acute and severe respiratory illnesses, along with influenza‐like illness (ILI).^10^ This programme is operated by the National Public Health Laboratories and National Influenza Centre, supported by the Omani Ministry of Health, World Health Organisation (WHO) and US Centre for Disease Control, along with other entities providing technical support. During the COVID‐19 pandemic, this network was placed under severe strain due to the surge in laboratory testing and surveillance needed to monitor SARS‐CoV‐2 transmission, which resulted in delays in testing for other pathogens; however, the network response was highly effective, with Oman National Public Health Laboratories being declared a reference centre for sequencing and genomic surveillance in the Eastern Mediterranean region by WHO in 2022. Oman provided laboratory and technical support to neighbouring countries who lacked either reagents, technical or logistical supplies to meet demand in their own countries, as well as technical expertise for genomics to the region.

Despite the efforts taken during COVID‐19, the Omani team remained highly concerned about the spread of other respiratory pathogens circulating in Oman. It was observed in other countries, such as Australia, that the measures taken to combat SARS‐CoV‐2 circulation prior to widespread vaccination, such as lockdowns or quarantine measures, border closures, handwashing and face masks, improved ventilation and limiting large crowd gatherings,^11^ resulted in concurrent reduction in the spread of viruses such as influenza A and B.^12^ To continually provide the latest information supporting public health interventions, Oman undertook a differential testing programme to identify respiratory pathogens circulating in the country, looking at 22 common pathogens associated with severe outcomes. Through this study, the aim is to inform public health policy and recommend best practices for continued surveillance and treatment of respiratory pathogens in circulation, with the goal of preventing spillover or outbreak events from becoming epidemics or pandemics in future.

## 2 METHODS

### 2.1 Cohort selection

Samples were taken from patients meeting case definition for severe acute respiratory infection (SARI), specifically a history of fever or a measured fever >38°C, cough with an onset within 10 days requiring hospitalisation. Samples were tested for both influenza and SARS‐CoV‐2 in sentinel sites. If the patient requires treatment in the ICU or high dependency unit at any site (sentinel and non‐sentinel), then the test panel is expanded to include other respiratory viruses (RVP) according to Omani medical standards.

### 2.2 Molecular testing with the respiratory viral pathogens panel (RVP)

Samples were transported to the Oman Central Public Health Laboratory, where they were inactivated and extracted using mainly MagMAX kit with KingFisher (Thermo Fisher) automated extraction system. Other extraction kits also used are Liferiver (Liferiver Biotech) and QIASymphony (Qiagen) automated platforms according to availability. For all platforms, manufacturer's instructions were followed. All kits were procured by the Ministry of Health for use by the National Public Health Laboratory. Extracted nucleic acid was then tested using the RVP assay following the manufacturer's instructions. FTD was the main assay used, sometimes other assays were also used like Viasure Respiratory Panel Kit (Certest Biotec) depending on availability. For comparison, percentage positivity was used, referring to the percentage of positive samples identified in the cohort when a specific pathogen was analysed. Significance was calculated using paired *t*‐tests comparing the percentage increase of infection year on year, with *P* values listed in the figure legends.

## 3 RESULTS

### 3.1 Differential molecular testing of SARS‐CoV‐2‐negative patients identified severe respiratory pathogens in cases meeting SARI case definition, with a significant drop in cases identified during COVID‐19

**FIGURE 1:**
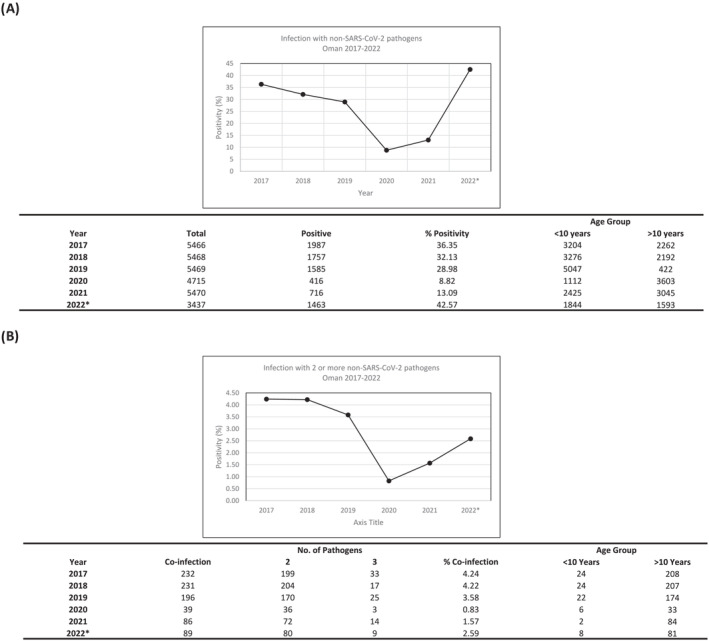
Respiratory infection rates with pathogens other than SARS‐CoV‐2 were generally lower during the peak of the COVID‐19 pandemic in 2020/21. (A) A cohort of patients who tested negative for SARS‐CoV‐2 were tested for infection with other common respiratory pathogens using a respiratory testing panel. (B) Infection with two or more pathogens was also assessed to investigate possible cases of sequential or co‐infection in patients presenting with SARI.

The RVP panel allows for comprehensive analysis of common respiratory viruses (Figure 1). Over the 6 years of the study, over 30 k samples were assessed, with a peak of ~5470 samples in each of 2017–2019, before a slight drop to 4715 samples in 2020, likely due to the high prevalence of SARS‐CoV‐2 infection, which peaked during 2020. Sample numbers rebounded to 5470 in 2021 and were on target to reach the same number in 2022, with 3437 samples tested up to September. Positivity for non‐SARS‐CoV‐2 pathogens was broadly comparable from 2017 to 2019, ranging from 30% in 2019 to 36% earlier in 2017 and 32% in 2018.

In comparison, 2020 showed a steep decline in respiratory infection to 9% with a slight increase to 13.09% in 2021, a 340% decrease and 230% decrease compared to 2019. An assessment of the media at this time indicates that this was during the stage when most stringent national infection control measures were in place for SARS‐CoV‐2. A closer analysis of the data showed that most cases identified in 2021 came in the second half of the year, between July and December (68%), with the borders opening to COVID‐19 vaccinated travellers in September 2021 possibly contributing to the increased infection numbers.

In 2022, the prevalence of respiratory pathogens has rebounded to 43% in SARI cases, an increase of 325% compared to 2021, and slightly higher than pre‐COVID‐19 restrictions. This has been driven largely by an increase in the prevalence of influenza, RSV and human rhinovirus, which will be investigated further in the next section, but may indicate that a reduction in humoral and herd immunity to respiratory pathogens due to the low numbers of infection in previous years, as has been observed in other countries.^13^

### 3.2 An assessment of individual respiratory pathogens identifies peaks and troughs of infection pre‐, during‐ and post COVID‐19 restrictions

**FIGURE 2:**
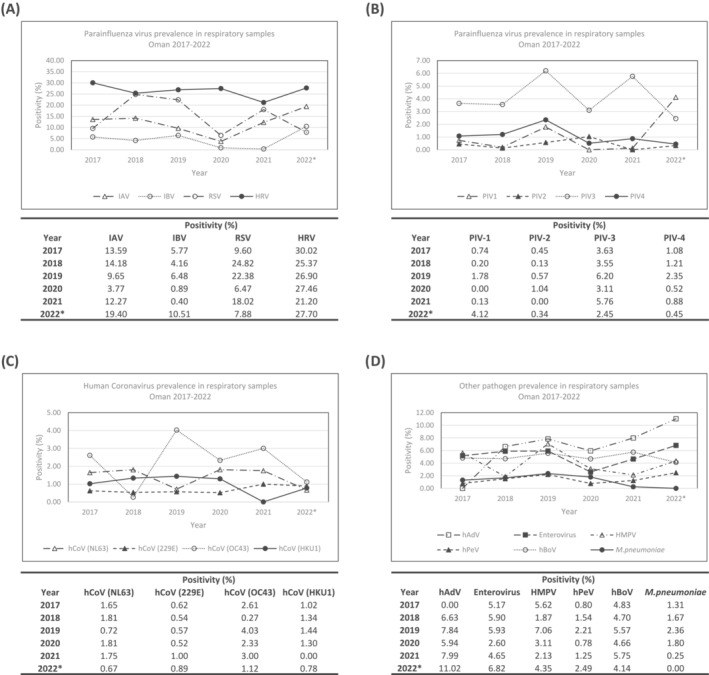
Differential testing of viral and bacterial pathogens in respiratory samples from across Oman between 2017 and 2022. Using molecular diagnostics, samples testing negative for SARS‐CoV‐2 were tested for (A) influenza A, influenza B, respiratory syncytial virus (RSV) and human rhinovirus (HRV), (B) human parainfluenza virus 1–4 (PIV1–4), (C) human coronaviruses (hCov) NL63, 229E, OC43 and HKU1, and (D) miscellaneous other pathogens including human adenovirus (hAdV), enterovirus, human metapneumovirus (HMPV), human parenchovirus (hPeV), human bocavirus (hBoV) and mycobacterium pneumoniae (*Mycoplasma pneumoniae*).

To assess whether the increases and decreases in infection were driven by changes in any pathogen between 2017 and 2022 (Figure 2), the individual results for each pathogen were grouped and analysed on a yearly basis. Influenza A and B showed differing profiles of prevalence, with IAV dropping consistently from 2018 to 2020 (14%–4%, a reduction of 375%). In contrast, IBV remained elevated in 2019 at 6% compared to 2018 at 4%, an increase of 155%, indicating that there may have been a circulating variant in this time frame that spread more widely than IAV. However, by 2020, this had dropped to 0.4% of cases, a decrease of 1620%, indicating that infection control measures may have contributed to reducing the spread of this virus. WHO‐recommended vaccination schedules differ between hemispheres, with the vaccination programme running through summer in Oman, to coincide with the Hajj pilgrimage to Mecca, which brings a large, mobile population to the region.

A concerning rebound of infection is observed in 2021 and 2022, with both IAV and IBV increasing to well above pre‐COVID‐19 levels, at 19% and 11%, respectively, increases of nearly 200% for both compared to pre‐pandemic levels. RSV and human rhinovirus showed similar profiles of infection to influenza, with both showing drops in infection between 2019/2020 before rebounding in 2021/2022, with a similarly concerning increase in 2022 to above pre‐pandemic levels. These viruses are all highly transmissible and require careful monitoring in the coming months to ensure they do not surge due to limited exposure in previous years.

In comparison, human coronavirus prevalence remained largely stable from 2017 to 2022, despite the surge in cases of SARS‐CoV‐2, or potentially due to this surge in cases. Prior to SARS‐CoV‐2, the infection mechanisms of coronaviruses were not widely studied, and it is possible that they may use similar entry pathways to SARS‐CoV‐2, and the limited transmission may be due to competition between these viruses and the newer emerged virus, though studies are yet to conclude any linkages. Studies are ongoing into this^14^ as well as immune profiling to see whether vaccination is protective against a broad panel of coronaviruses.

Parainfluenza virus has remained stable across the study duration, except for a surge in PIV‐3 and PIV‐4 cases in 2019. An assessment of the collection dates of the samples showed that 48% of positive samples were collected in January of 2019, which may indicate that there was a regional or localised surge in cases. This dropped significantly by 2020 and has remained stable at approximately 4%–6% in subsequent years. An alarming surge in cases PIV‐1 cases has been seen in 2022, up from 0.13% to 4%. There is no discernible pattern or month where cases have surged, indicating that there may be circulating strains that need close monitoring in coming months.

Of the remaining viruses and pathogens assessed, human adenovirus remained largely stable through the pandemic but has shown an increase in prevalence of 137% in 2022 compared to previous years. This is concerning with the recent link between adenovirus and liver failure in children^15^ and should be monitored. Similarly human metapneumovirus and parechoviruses have increased by over 200% though have not reached pre‐COVID‐19 levels, so this may be a return to normal infection levels due to relaxing of infection controls after the pandemic.

## 4 DISCUSSION

SARI remains a significant and ongoing public health concern in Oman, despite the decrease in SARS‐CoV‐2 positivity, which has been observed in recent times. While the COVID‐19 pandemic is not over, surveillance efforts, which were largely focused on SARS‐CoV‐2 for the past 3 years, are being redirected, looking to integrate SARS‐CoV‐2 with existing SARI/ILI surveillance mechanisms. Integrated disease surveillance and response (IDSR) is not a new concept, being widely used by CDC, WHO and other health bodies across Africa.^16^ The heavy investment in equipment, training and expertise that has been gathered during the COVID‐19 pandemic, however, has presented a unique opportunity to both expand and integrate surveillance mechanisms including molecular diagnostics to develop a more holistic platform to prevent the next pandemic from occurring.

Oman has maintained a strong surveillance system throughout the COVID‐19 pandemic, both for SARS‐CoV‐2 and other respiratory pathogens, though this placed extreme pressure on laboratory and health infrastructure in the country. Previous investment in data sharing and information technology platforms allowed for the rapid and stable implementation of reporting mechanisms for infection, which were capitalised on during the pandemic and will continue to be developed for other pathogens moving forward.

The results of this study are largely consistent with other national surveys into the prevalence of respiratory pathogens pre‐, during and post‐COVID‐19. The implementation of control measures for SARS‐CoV‐2, a respiratory pathogen, had a significant impact on the spread of other respiratory pathogens.^12^ However, the rebound effect of this is a reduction in herd immunity to these pathogens once the restrictions were relaxed.^17^

Influenza is a key and clear example of this effect. Influenza infection was low both in Oman, the United States and other countries with influenza surveillance programmes, in spite of high levels of testing, indicating that there was no specific influenza season, which is normally observed globally during autumn/winter months. Indeed, the CDC reported most positive infections of influenza A were H3N2 and influenza B were Victoria strain, which was nearly identical to the previous year, indicating very little recombinant or novel variants were circulating during the pandemic, while cases were also seen in Bangladesh and Cambodia in early 2020.^18^

In contrast, in 2021/2022, the levels rebounded towards pre‐pandemic levels, and while most circulating strains remained vaccine strains (H3N2 and Victoria strains), the season lasted several months longer than usual. While this is partially due to the season starting earlier than usual, the length is concerning leading into the next season, with less time to develop immunity. It is critical that vaccination efforts be maintained in coming months, to ensure that any reductions in herd immunity are combated effectively to prevent an influenza season with severe clinical impact from occurring.^15^

As discussed earlier, RSV is another concern, particularly for children, the elderly and immunocompromised individuals. While the levels have not rebounded entirely to pre‐pandemic levels, an analysis of the circulating variants will be carried out to help guide vaccination policies in future, as several vaccines have reached phase 3 testing and may be available in coming years.^19^ In comparison, human rhinovirus showed no significant reduction in circulation at any stage over the past 6 years. This could be due to the limited efficacy of masks in preventing transmission of rhinovirus, as reported in studies in 2022 and 2023.^20^ While rhinovirus may have a lower morbidity than other viruses in this study, the level of impact on health systems is significant and remains a concern moving forward, as there are no vaccines approaching release.

The gradual increase in the presence of human adenovirus in respiratory samples is of concern primarily because adenovirus is a widely prevalent virus in humans and rarely has a pathological effect. This is one of the main reasons it is used in novel vaccines and therapeutic delivery vectors, as it is highly efficient at cell entry and dissemination.^21^ However, there is increasing evidence that co‐infection with adenovirus and other pathogens can exacerbate existing conditions, including liver failure in children. ECDC reported an alarming increase in children dying from liver failure^22^ that was later associated with co‐infection with adenovirus. While this was due to an enteric strain of adenovirus, the risk of co‐infection between adenovirus and other circulating pathogens is concerning. The steady increase from 6% positivity to 11% over 3 years bears monitoring, particularly for non‐respiratory or systemic issues that may be associated with the virus.

Overall, the ongoing SARI surveillance system in Oman has clearly identified surges and waves of infection with pathogens other than COVID‐19, demonstrating the risks associated with focusing on a single pathogen, even during a pandemic. Respiratory pathogens present the highest risk to the population, and while the data have shown that spread can be mitigated by infection control measures, this control needs to be maintained; otherwise, the pathogens re‐emerge, often with higher frequency than before the control measures were implemented, due to loss of immune memory^17^, ^18^ and herd immunity. Continual, integrated disease surveillance is a key to guiding public health interventions and decision making, allowing resources to be mobilised effectively to prevent the next outbreak from becoming an epidemic or pandemic in the future.

## AUTHOR CONTRIBUTIONS

**Hanan al‐Kindi:** Conceptualization; funding acquisition; project administration; supervision; writing—review and editing. **Luke William Meredith:** Writing—original draft; writing—review and editing. **Amina Al‐Jardani:** Conceptualization; funding acquisition; project administration; supervision; writing—original draft; writing—review and editing. **Fatima Sajina:** Data curation; formal analysis; methodology; writing ‐ original draft; writing—review and editing. **Intisar al‐Shukri:** Data curation; formal analysis; methodology; writing—original draft; writing—review and editing. **Rehan al‐Haji:** Data curation; formal analysis; methodology; writing—original draft; writing—review and editing. **Fatma al‐Yaquobi:** Data curation; investigation; methodology; supervision; writing—original draft; writing—review and editing. **Adil al‐Wahaibi:** Formal analysis; investigation; methodology; project administration; supervision; writing—original draft; writing—review and editing. **Amal al‐Maani:** Formal analysis; investigation; methodology; supervision; writing—original draft; writing—review and editing.

## CONFLICT INTEREST STATEMENT

The authors declare no conflicts of interest.

## Data Availability

All data generated in this manuscript are the property of the Omani Ministry of Health and Central Public Health Laboratory. Requests can be made directly for access to the raw data.
